# Characteristics of antibiotic exposures for surgical procedures prior to *Clostridioides difficile* diagnosis—Minnesota, 2018

**DOI:** 10.1017/ash.2022.105

**Published:** 2022-05-16

**Authors:** Paige D’Heilly, Amanda Beaudoin, Davis Melin, Stacy Holzbauer

## Abstract

**Background:** *Clostridioides difficile* infection (CDI) is the leading cause of healthcare-associated diarrhea. Significant risk factors for CDI include antibiotic use and healthcare exposure. Antibiotics are often administered before, during and/or after surgery to prevent postsurgical infection. The contribution of surgery-related antibiotics to the overall CDI burden has not been well described, and assessment of the appropriateness of surgical antibiotic use is complicated by complex clinical guidelines. We have described surgical antibiotic prophylaxis history among adult with CDI in Minnesota in 2018. **Method:** The Minnesota Department of Health (MDH) performs 5-county active population- and laboratory-based CDI surveillance as a CDC Emerging Infections Program site. Incident CDI was defined as stool positive for *C. difficile* by toxin or molecular assay from a person aged ≥18 years with no positive test in the preceding 8 weeks. History of CDI was defined as having had a previous CDI episode in the 2009–2018 surveillance data set. Medical records were reviewed for 12 weeks prior to incident CDI test date to identify antibiotic prescriptions. Antibiotics with documented indication for surgical-site infection prevention or surgical prophylaxis were classified as “surgical antibiotic prophylaxis” (SPPX). SPPX type (eg, intraoperative, postoperative), appropriateness of SPPX, and clinical guideline adherence were not assessed. **Results:** During 2018, 812 incident CDIs were reported to MDH among 736 patients. SPPX preceded 84 (10.3%) cases, non-SPPX antibiotic use preceded 465 cases (57.3%), and 263 cases (32.4%) had no documented prior antibiotic use. The median age of incident CDIs with preceding SPPX was 68 years (IQR, 54–79.5). In 25 incident CDI cases with preceding SPPX (29.8%), there were no other antibiotic exposures. Among incident CDIs with preceding SPPX, 11 (13.1%) had >1 surgery event with SPPX. Prior CDI was identified for 13 (15.7%) with SPPX. Among 99 procedures with preceding SPPX, orthopedic surgeries (n = 27, 27.3%), gastrointestinal surgeries (n = 26, 26.3%), and cardiovascular surgeries (n = 22, 22.2%) were most common. In total 18 SPPX prescriptions (18.2%) originated in outpatient settings. SPPX drugs included cefazolin (n = 67, 67.7%), ceftriaxone (n = 7, 7.1%), ertapenem (n = 6, 6.1%), and clindamycin (n = 6, 6.1%). Median SPPX duration was 1 day (IQR, 1–2), and the median number days between surgery and specimen collection date was 19 (IQR, 7–49). **Conclusions:** Antibiotic stewardship programs should assess surgical prescribing, including in outpatient centers. Even short antibiotic duration for surgery could put patients at risk for CDI. More data are needed to evaluate the appropriateness of SPPX prescribing and to describe the impact of SPPX on CDI.

**Funding:** None

**Disclosures:** None

